# Relapsed Classical Hodgkin Lymphoma in Pregnancy in Two Patients Managed With a Multidisciplinary Approach

**DOI:** 10.1155/crom/3286507

**Published:** 2025-12-01

**Authors:** Minhal Zaidi, Amna Naqvi, Jacqueline Rios, Meera Khosla, Hala Hassanain, Noah Giese, Ethan A. Burns, Hanh Mai, Carrie Yuen, Shilpan Shah, Siddhartha Ganguly, Sai Ravi Pingali

**Affiliations:** ^1^Department of Academic Medicine, Houston Methodist Hospital, Houston, Texas, USA; ^2^Houston Methodist Neal Cancer Center, Houston Methodist Hospital, Houston, Texas, USA

## Abstract

Relapsed or refractory (r/r) classical Hodgkin lymphoma (cHL) during pregnancy is rare, and management is often complex. The following two cases of r/r cHL during pregnancy highlight management considerations and outcomes in this unique patient population. The first patient with Stage IIIB cHL achieved a complete response (CR) with ABVD (doxorubicin, bleomycin, vinblastine, and dacarbazine) prior to pregnancy but relapsed at 10 weeks of gestation. She was maintained with methylprednisolone and vinblastine with no complications until she underwent cesarean section at 34 weeks. The second patient, diagnosed with Stage IIIb cHL, achieved CR after five cycles of ABVD and one cycle of AVD. She began consolidation radiation but halted treatment after two cycles upon discovering an intrauterine twin pregnancy. At 10 weeks of gestation, she experienced a relapse of her disease. The patient received one cycle of vinblastine and methylprednisolone weekly. At 31 weeks, the patient underwent an elective cesarean section. After delivery, both patients underwent ICE (ifosfamide, carboplatin, and etoposide), followed by consolidation with autologous hematopoietic cell transplantation (auto-HCT). These cases highlight the balance needed to maintain control of disease to allow a safe and uneventful pregnancy.

## 1. Introduction

Classical Hodgkin lymphoma (cHL) comprises 6% of all pregnancy-related cancers [1]. Pregnant women with relapsed or refractory cHL require careful consideration of disease burden, gestational age, timing of relapse relative to prior treatments, and the safety profile of available therapies. In the second and third trimesters, chemotherapy regimens such as ABVD (doxorubicin, bleomycin, vinblastine, dacarbazine) have been employed, with evidence suggesting their relative safety during these periods. However, the optimal management strategy remains a subject of ongoing clinical investigation [2]. In this case series, two instances of relapsed cHL during pregnancy are discussed, highlighting the complexities of treatment decision-making and the importance of multidisciplinary collaboration in managing such cases.

## 2. Case Presentations

### 2.1. Case 1

A 25-year-old female with a history of Stage IIIB cHL (nodular sclerosing subtype) achieved complete remission following six cycles of ABVD chemotherapy in 2016. Eighteen months later, she presented with rapidly enlarging lymphadenopathy located in her neck, chest, and pelvis. CT imaging confirmed extensive adenopathy and an unanticipated pregnancy. Her care was delayed due to loss to follow-up, and she re-presented at Gestational Week 23, and excisional lymph node biopsy confirmed relapsed cHL.

She was treated with four cycles of vinblastine and methylprednisolone during pregnancy. Vinblastine was administered every 2 weeks at a dose comparable to the standard ABVD schedule, for a total of four doses. Methylprednisolone 125 mg was administered with each vinblastine treatment, and the final dose was administered 4 weeks prior to cesarean delivery at 34 weeks. The newborn initially required CPAP in the NICU but was successfully weaned off oxygen.

At 4 weeks postpartum, PET imaging revealed extensive disease in the neck, chest, abdomen, and pelvis. She subsequently received three cycles of ICE chemotherapy, achieving an excellent partial response. This was followed by auto-HCT with a BEAM-R conditioning regimen. Twenty-one days after receiving auto-HCT, the patient was lost to follow-up and did not get follow-up labs or imaging. She presented 15 months later with lymphadenopathy in the left inguinal region, and biopsy confirmed cHL. She underwent involved field radiation therapy and completed one cycle of brentuximab and nivolumab. When she returned for her second cycle, shortly after the infusion of brentuximab began, she developed orthostatic hypotension, prompting discontinuation of the infusion. Imaging at that time including a CT of the head, chest, abdomen, and pelvis showed no evidence of active disease. Following this event, she was lost to follow-up and did not complete further treatment or undergo any additional evaluation.

### 2.2. Case 2

A 33-year-old female with a history of Stage IIIa cHL completed five cycles of ABVD and one cycle of AVD, with bleomycin discontinued due to bleomycin-induced lung injury in 2016. This was followed by consolidation radiation therapy to the anterior mediastinum. After completing two cycles of consolidation radiation, the patient noticed irregular menses and presented to her OB-GYN for contraception counseling. A pelvic ultrasound revealed a 5-week intrauterine twin pregnancy. This was 3 months after her last chemotherapy, and given her recent radiation exposure, the potential effects on fetal development were uncertain, and options including termination were discussed. She elected to continue the pregnancy.

At week 15 of gestation, she experienced intrauterine fetal demise of one twin, suspected to be due to Trisomy 13. Unfortunately, at 22 weeks of gestation, she developed progressive shortness of breath and lymphadenopathy. Approximately 7 months after her last chemotherapy, she was found to have a relapse of cHL with a CT scan showing enlarging lymphadenopathy and an axillary lymph node biopsy consistent with cHL.

After a multidisciplinary discussion, she was initiated on methylprednisolone weekly. A follow-up CT revealed that the anterior mediastinal mass had slightly decreased in size, but there was extensive new and enlarging confluent lymphadenopathy throughout the chest, compatible with progressive disease. Vinblastine was started to help control her disease progression. However, after the first cycle, she experienced an extravasation of vinblastine in her arm. Because of this complication, the patient decided not to continue with further vinblastine treatments. At 32 weeks of gestation, she underwent a cesarean section. The newborn initially required CPAP support for hypoxia and was transferred to the NICU, where respiratory support was gradually weaned off.

Following delivery, she received three cycles of salvage chemotherapy with ICE followed by auto-HCT. A follow-up PET scan revealed recurrent lymphoma in the anterior/inferior mediastinum with sternal invasion. She was treated with 20 doses of nivolumab, after which PET/CT showed complete remission.

## 3. Discussion

r/r cHL arising during pregnancy presents a complex clinical challenge. Management is influenced by various factors, including the extent of disease burden, the specific trimester during which the relapse occurs, and the timing of the relapse in relation to prior treatments the patient has received. More importantly, the range of therapeutic options that are safe to administer without causing harm to the developing fetus requires careful consideration. Much of the management of relapsed or refractory cHL during pregnancy is derived from retrospective studies and case reports, with the majority from 1976 to 1992. Evens et al. compiled 40 women treated with cHL and found 3-year progression-free survival (PFS) and overall survival (OS) of 85% and 97%, respectively. To our knowledge, this is the only series reporting management of relapsed cHL patients [3]. These two cases highlight the unique challenges of managing relapsed cHL during pregnancy, and treatments were tailored to the clinical situation. Both patients initially achieved remission with ABVD, complicated by bleomycin-induced pulmonary toxicity, and subsequently experienced relapse during pregnancy. In both cases, vinblastine and corticosteroids were used as temporizing therapy.

The postpartum courses differed in each case. In the first case, the patient experienced delays in diagnosis and interruptions in care, resulting in multiple relapses and incomplete therapy. In the second case, the patient remained engaged in care, underwent timely salvage chemotherapy, autologous stem cell transplantation, and immunotherapy, ultimately achieving complete remission.

The trimester during which the cHL diagnosis is made greatly influences treatment decisions. If cHL is diagnosed in the first trimester, treatment is typically deferred until the second or third trimester [2]. However, if symptoms necessitate the need for treatment, vinblastine is typically favored as the first-line therapy. Vinblastine has a lower risk of fetal toxicity due to lower placental transfer compared to other antineoplastic agents. Using vinblastine as monotherapy can help delay treatment until the second or third trimester [4]. Multiple studies have reported that vinblastine monotherapy is safe during the second and third trimester. However, there have been documented teratogenic complications associated with use in the first trimester due to critical organogenesis [4]. Corticosteroids are used for temporary disease control until standard chemotherapy regimens can be initiated [5]. Moreover, utilizing therapies particularly after patients have relapsed is an important consideration. In both cases here, vinblastine was used to maintain disease control until delivery.

In the second or third trimester, the standard chemotherapy regimen includes ABVD. Studies have shown that ABVD chemotherapy during the second and third trimesters is associated with favorable maternal and fetal outcomes, with no significant increase in congenital anomalies or long-term developmental issues. Maternal response and prognosis are comparable to nonpregnant patients, supporting the use of ABVD when treatment cannot be delayed [6]. Due to concerns about pulmonary toxicity, bleomycin is often omitted from treatment regimens. Nivolumab and brentuximab are not recommended during pregnancy due to risks of fetal harm shown in animal studies, including miscarriage and birth defects. Due to rare immune-related neonatal adverse events, checkpoint inhibitors should be avoided in pregnant individuals [7]. A multidisciplinary approach to treating r/r cHL during pregnancy is essential. An early cesarean section may sometimes be necessary to begin chemotherapy sooner.

Radiation therapy is generally avoided during pregnancy for lymphoma due to risks of fetal birth defects, especially in the first trimester. If absolutely necessary, it may be used with fetal shielding after careful multidisciplinary review, but this is rare. Most cases are managed with chemotherapy or delayed until after delivery [5]. G-CSF is not routinely used during pregnancy but may be given to treat severe neutropenia. Available data suggest it does not increase birth defect risks, but it should only be used when clearly needed after careful risk–benefit evaluation [8].

Given the significant reproductive risks associated with the use of chemotherapy and radiation, contraceptive counseling in women of reproductive age is essential. Effective contraceptive counseling for patients with cancer must be tailored to their specific cancer type and disease stage [9, 10].

When staging or assessing lymphoma during pregnancy, imaging should prioritize fetal safety. Ultrasound and MRI without contrast are preferred. CT and PET/CT scans, which involve radiation, are usually avoided. Chest x-rays with abdominal shielding are also considered safe when needed [4].

## 4. Conclusion

These cases emphasize the importance of multidisciplinary management and patient-specific approaches, early recognition of relapse, and continuity of care to optimize both maternal and fetal outcomes in the setting of Hodgkin lymphoma during pregnancy.

## Data Availability

Data sharing is not applicable to this article as no datasets were generated or analyzed during the current study.
